# Tunable Mid-Infrared Interband Emission from Tensile-Strained InGaAs Quantum Dots

**DOI:** 10.1021/acsnano.2c08985

**Published:** 2023-01-17

**Authors:** Kevin D. Vallejo, Carlos I. Cabrera-Perdomo, Trent A. Garrett, Madison D. Drake, Baolai Liang, Kevin A. Grossklaus, Paul J. Simmonds

**Affiliations:** † Condensed Matter and Materials Physics, Idaho National Laboratory, Idaho Falls, Idaho83415, United States; ‡ Unidad Académica de Ciencia y Tecnología de la Luz y la Materia, Universidad Autónoma de Zacatecas, 98160Zacatecas, Zac., Mexico; ¶ Micron School of Materials Science & Engineering, Boise State University, Boise, Idaho83725, United States; § California NanoSystems Institute, University of California Los Angeles, Los Angeles, California90095, United States; ∥ Department of Electrical and Computer Engineering, Tufts University, Medford, Massachusetts02155United States; ⊥ Department of Physics, Boise State University, Boise, Idaho83725, United States

**Keywords:** quantum dots, molecular beam epitaxy, mid-infrared, tensile strain, self-assembly

## Abstract

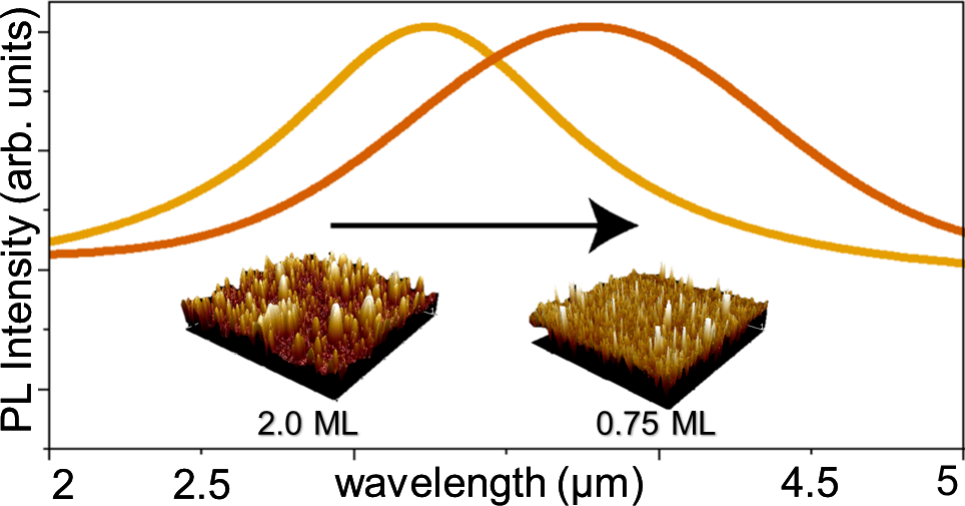

We demonstrate the ability to tailor self-assembled growth of In_0.5_Ga_0.5_As quantum dots (QDs) on GaSb(111)A surfaces by molecular beam epitaxy. Spontaneous formation via the Volmer–Weber growth mode produces QDs with excellent structural and optical quality. By harnessing tensile strain to reduce their band gap energy, these QDs are characterized by light emission that extends into the midwave infrared wavelength range of 3.2–3.9 μm (0.318–0.388 eV). As we increase QD size, we can tune the band alignment from type-III to type-II, where light emission occurs due to interband recombination between quantum confined electrons in the InGaAs QDs and holes in the GaSb barriers. Of particular interest is an unusual blue-shift in emission wavelength with increasing QD size, which we attribute to the incorporation of Sb into the InGaAs QDs from the GaSb barriers. By expanding this approach to produce tensile-strained QDs from other narrow band gap semiconductors, we anticipate the development of a range of highly tunable mid-infrared light sources.

## Introduction

Interest in III–V semiconductors grown on surfaces other than the traditional (001) plane has increased in recent years, with (111)-oriented materials emerging as an area of particular interest. Although molecular beam epitaxy (MBE) on (111) surfaces is typically more challenging than on (001), researchers are identifying optimal growth conditions for (111)-oriented semiconductors, enabling us to begin taking advantage of their particular properties.^1,2^ The crystal symmetry of the (111) surface of III–V semiconductors facilitates their integration with dissimilar materials systems, including V_2_–VI_3_ topological insulators,^3,4^ IV–VI rocksalt semiconductors such as PbSe,^5^ and transition metal dichalcogenides.^6,7^ Transistors with a (111) orientation offer the potential advantage of ballistic electron transport in both Γ and *L* valleys, allowing them to overcome the density of states bottleneck.^8^

Self-assembled quantum dot (QD) nanostructures grown on (111) surfaces are also attracting attention. (111)-oriented QDs hold promise for quantum photonics applications such as photon entanglement.^9−11^ Tensile strain can be used to drive QD self-assembly on (111) surfaces. The specific combination of the tensile strain between the substrate and epilayer, and the dislocation kinetics on (111) surfaces, produces an energy landscape where QDs can nucleate and grow free from dislocations.^12^

What is more, the presence of residual tensile strain in these (111) QDs can be beneficial, in and of itself. Tensile strain serves to raise the light hole valence band above the heavy hole band,^13^ which could be useful for quantum transduction.^14^ Tensile strain also reduces the semiconductor band gap energy (*E*_g_) to red-shift the QD emission wavelength, an effect that is of interest for infrared (IR) optoelectronic applications.^10,15,16^ By using tensile strain to engineer the electronic structure of semiconductor QDs that already possess a narrow band gap, we may be able to push their emission deeper into the IR. Light-emitting diode (LED) and laser structures based on tensile strained QDs hence have the potential to be faster, cheaper, and less complicated to grow than quantum cascade structures for mid-IR applications.

To this end, in this paper we explore the self-assembly of tensile-strained In_0.5_Ga_0.5_As (hereafter InGaAs) QDs on GaSb(111)A by MBE and their resulting IR light emission properties. This is an unusually tunable QD system. Raising the Ga content widens the band gap of In_1–*x*_Ga_*x*_As, but simultaneously increases the tensile strain with the GaSb barrier layers, which acts to narrow *E*_g_. On top of this, we can control the energy of the QD confined states via their size. Finally, as we will demonstrate, the incorporation of Sb into the InGaAs QDs from the GaSb barriers also affects their emission wavelength. Understanding the relative strength of these competing effects will enable researchers to use the MBE growth parameters to tailor the properties of tensile-strained QDs for specific IR applications.

Previous studies have shown that InGaAs embedded in GaSb(001) forms quantum wells (QWs) with a type-II or -III band alignment where electrons are confined in the InGaAs, and holes localized in the GaSb.^17^ These QWs emit light in the 1770–2750 nm (0.45 to 0.7 eV) range as a function of the amount of InGaAs deposited, leading to the demonstration of laser devices.^18^ In the more highly tensile-strained GaAs/Ga(As)Sb(001) system, QDs form in addition to QWs, exhibiting light emission in the 1720–2170 nm (0.57 to 0.72 eV) range.^19,20^ However, dislocation-mediated relief of tensile strain in zinc-blende semiconductors is energetically far less favorable on (111) surfaces than on (001) surfaces.^12^ As a result, (111)-oriented QDs can accommodate higher tensile strains before the onset of dislocation formation than those on (001). This factor permits the growth of larger QDs that, considering quantum size effects alone, will emit deeper into the IR and hence provide additional control over emission wavelength.

Here, we investigate the self-assembly of InGaAs/GaSb(111)A QDs as a function of the MBE growth conditions. We demonstrate band-to-band recombination between electrons in the InGaAs QDs and holes in the GaSb barriers. Furthermore, the tensile strain of ∼4.1% means that the resulting light emission from the InGaAs/GaSb(111)A QDs at 77 K occurs at wavelengths ≥ 3.2 μm, i.e., at photon energies significantly below the InGaAs bulk band gap. We anticipate that tensile-strained QDs will form the basis for future optoelectronic devices designed for light emission in the mid-IR.

## Results/Discussion

### GaSb Homoepitaxy

**Figure 1:**
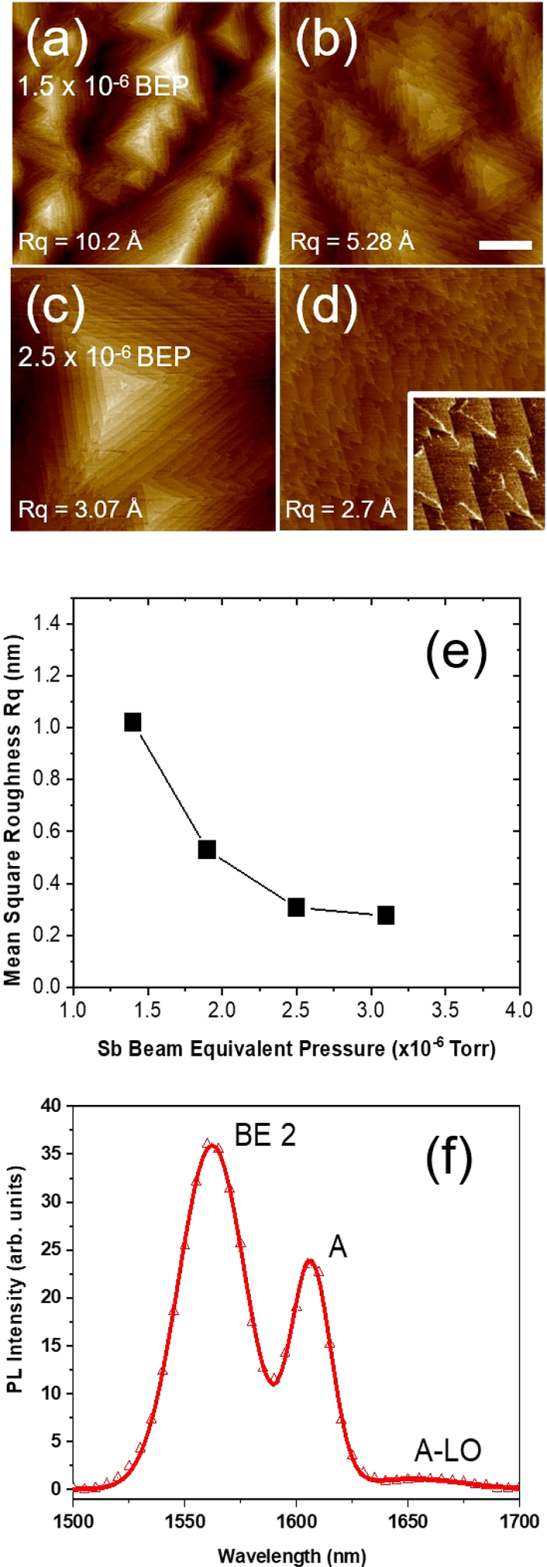
(a–d) 5 × 5 μm^2^ AFM images (*z*-scale = 6 nm, scale bar = 1 μm) showing GaSb(111)A surface evolution as we increase Sb_2_ BEP from 1.5 × 10^–6^ to 3.1 × 10^–6^ Torr. Inset to (d): a 1 × 1 μm^2^ closeup of the main image (*z*-scale = 1 nm). (e) GaSb(111)A surface Rq as a function of Sb_2_ BEP. (f) PL at 77 K of a homoepitaxial GaSb(111)A sample.

The starting point for this work was to obtain a smooth GaSb(111)A buffer layer, free of hillocks or other surface features that would be detrimental to QD growth (Figure 1). Under an Sb_2_ beam equivalent pressure (BEP) of 1.5 × 10^–6^ Torr, we see large pyramidal features across the surface (Figure 1(a)). Increasing the Sb_2_ BEP to 1.9 × 10^–6^ Torr flattens these structures significantly (Figure 1(b)), while a further increase to 2.5 × 10^–6^ Torr results in the appearance of isolated triangular islands that grow in the step-flow mode (Figure 1(c)). Finally, raising the Sb_2_ BEP to 3.1 × 10^–6^ Torr produces 2D step-flow growth with atomically flat, triangular terraces, ∼250 nm wide (Figure 1(d)). We calculate a root-mean-square roughness (Rq) for this surface of 2.76 Å averaged over a 25 μm^2^ area. The inset to Figure 1(d) reveals interesting linear features along the line of symmetry of several of these terraces, the origin of which will be the subject of future studies. Figure 1(e) shows the reduction in Rq of the GaSb(111)A surface as we raise the Sb_2_ BEP. To the best of our knowledge, values of Rq ≤ 3 Å represent the smoothest GaSb(111)A surfaces reported to date, offering an excellent starting surface for the growth of InGaAs QDs.

TEM images of homoepitaxial GaSb(111)A grown under these optimized conditions confirm their high-quality crystal structure; see for example the GaSb buffers beneath the QD layers in Figures 5 and 6. A 77 K photoluminescence (PL) analysis of the GaSb(111)A homoepitaxial films reveals three emission peaks at 1560, 1607, and 1652 nm (Figure 1(f)). We attribute these spectral features to three different recombination processes, respectively: exciton bound to a neutral acceptor (BE), residual acceptor (A), and longitudinal optical phonon replica of A (A-LO).^21^

**Figure 2:**
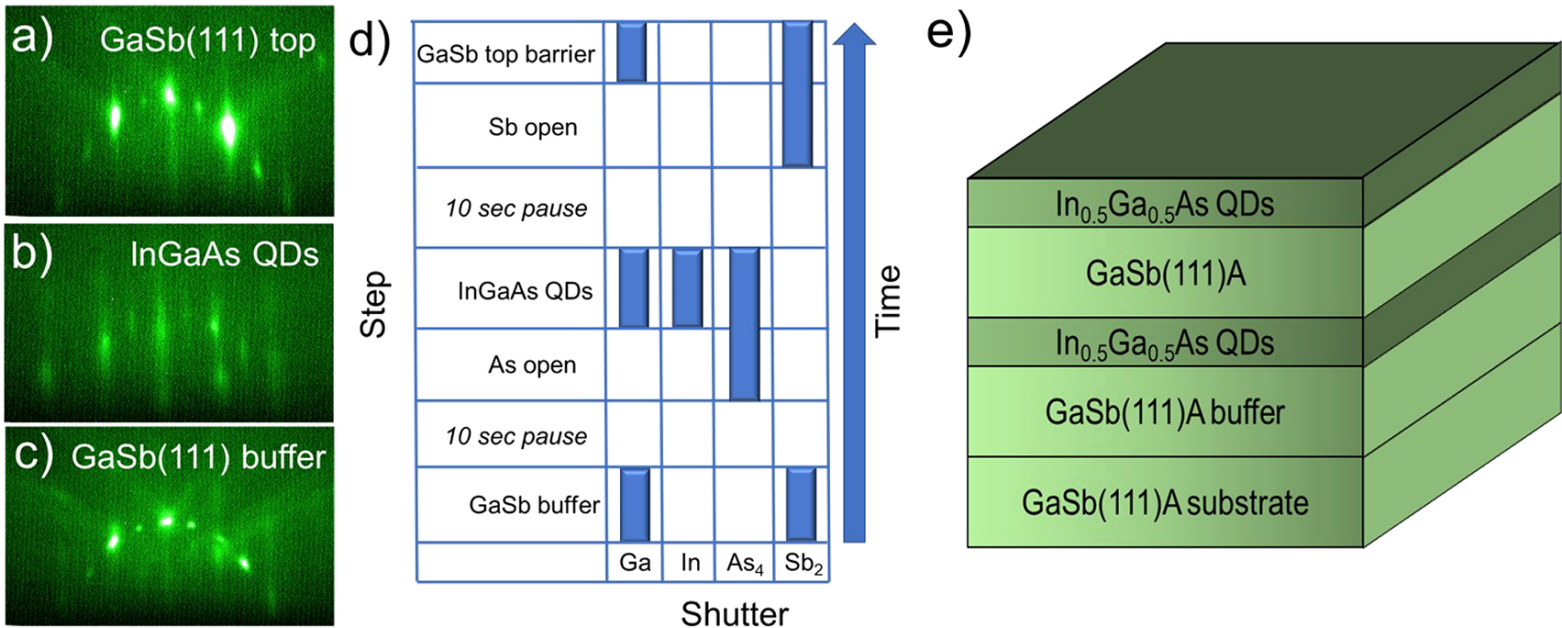
(a–c) RHEED sequence during InGaAs/GaSb(111)A sample growth. (d) Shutter sequence used during these growths, showing the 10 s pause step used when switching the group V flux from As_4_ to Sb_2_ after growth of the InGaAs QDs. (e) Schematic structure of all InGaAs QD samples studied.

**Figure 3:**
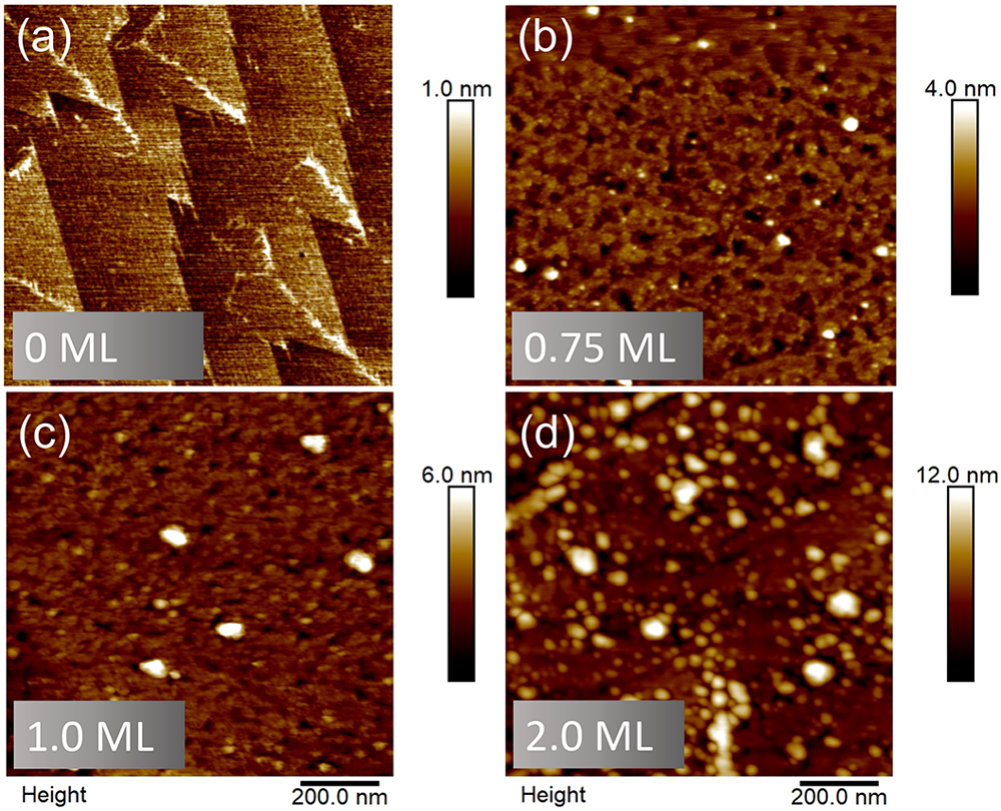
(a–d) 1 × 1 μm^2^ AFM images showing the surface morphology of 0–2 ML InGaAs deposited on GaSb(111)A at 450 °C at 0.4 ML/s, with a pause of 10 s.

**Figure 4:**
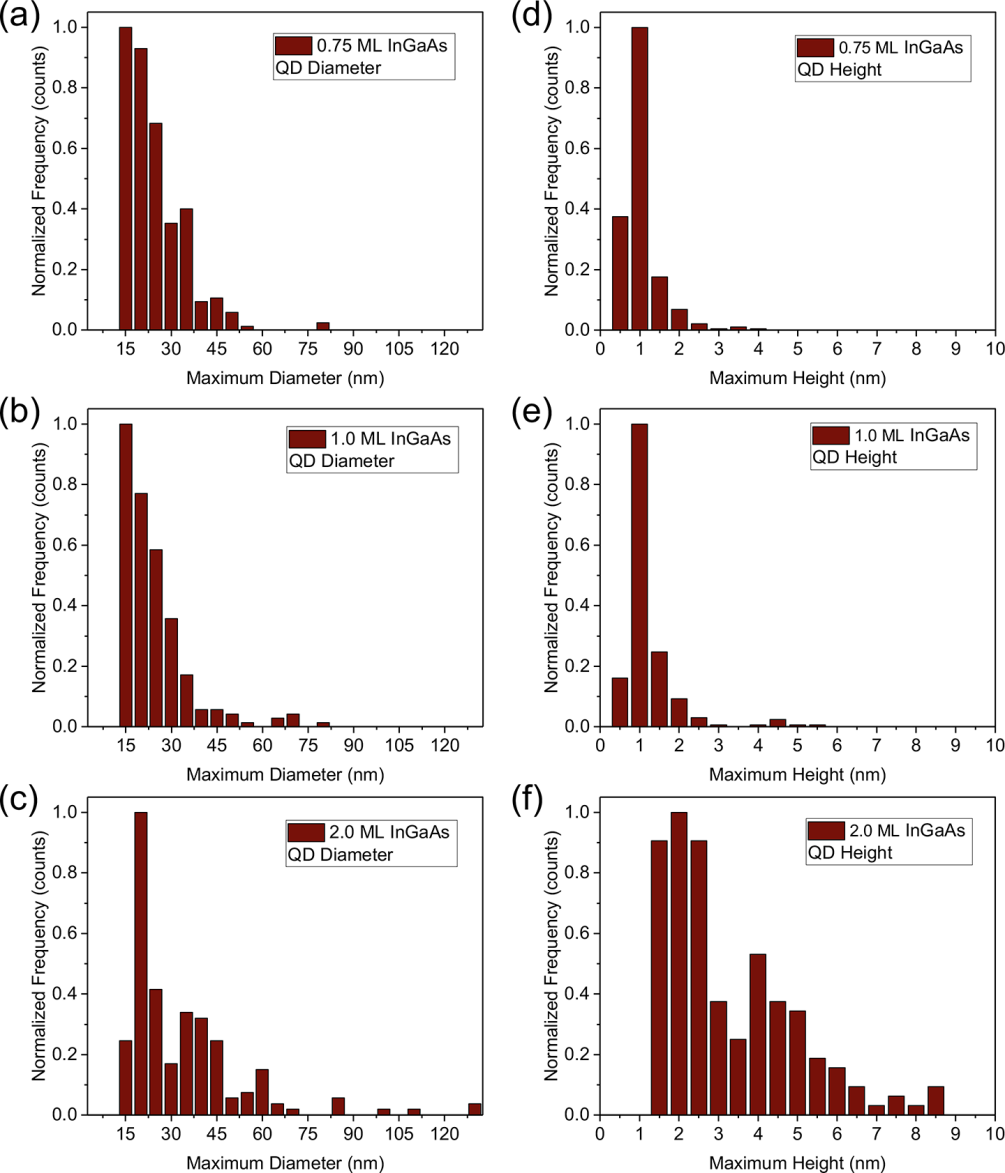
(a–c) Histograms showing distributions of InGaAs QD diameter for (a) 0.75 ML, (b) 1.0 ML, and (c) 2.0 ML InGaAs coverage. (d–f) Histograms showing distributions of InGaAs QD height for (d) 0.75 ML, (e) 1.0 ML, and (f) 2.0 ML InGaAs coverage. We see evidence of bimodal distributions for both diameter (c) and height (f) of the 2 ML QDs.

**Figure 5:**
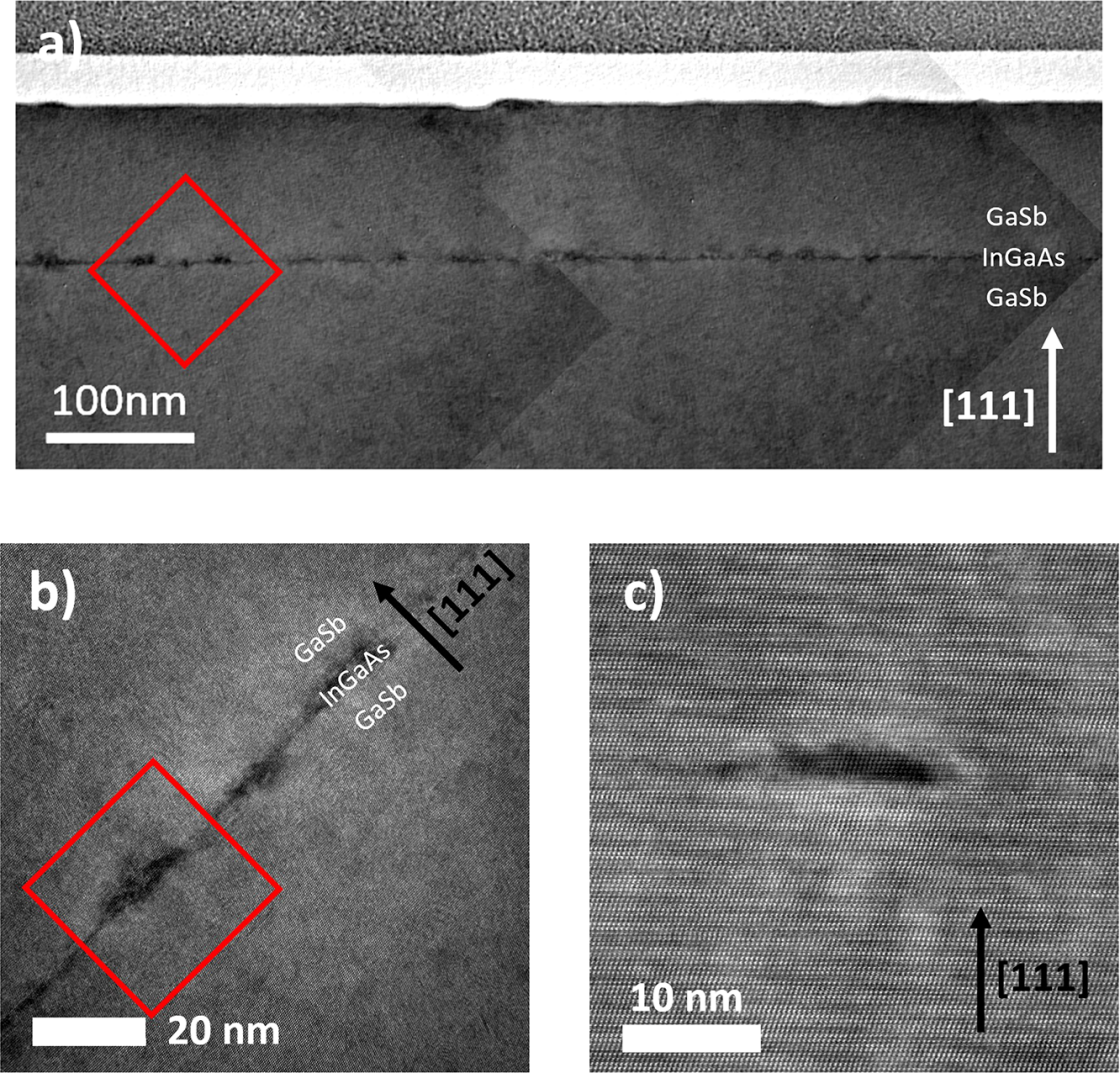
(a) BF TEM image montage of a 1 ML InGaAs QD layer surrounded by GaSb(111)A barriers, with a second layer of identical InGaAs QDs at the surface. (b) BF TEM image of several buried 1 ML InGaAs QDs corresponding to the red square in (a). (c) HAADF STEM image of the buried 1 ML InGaAs QD enclosed by the red square in (b).

**Figure 6:**
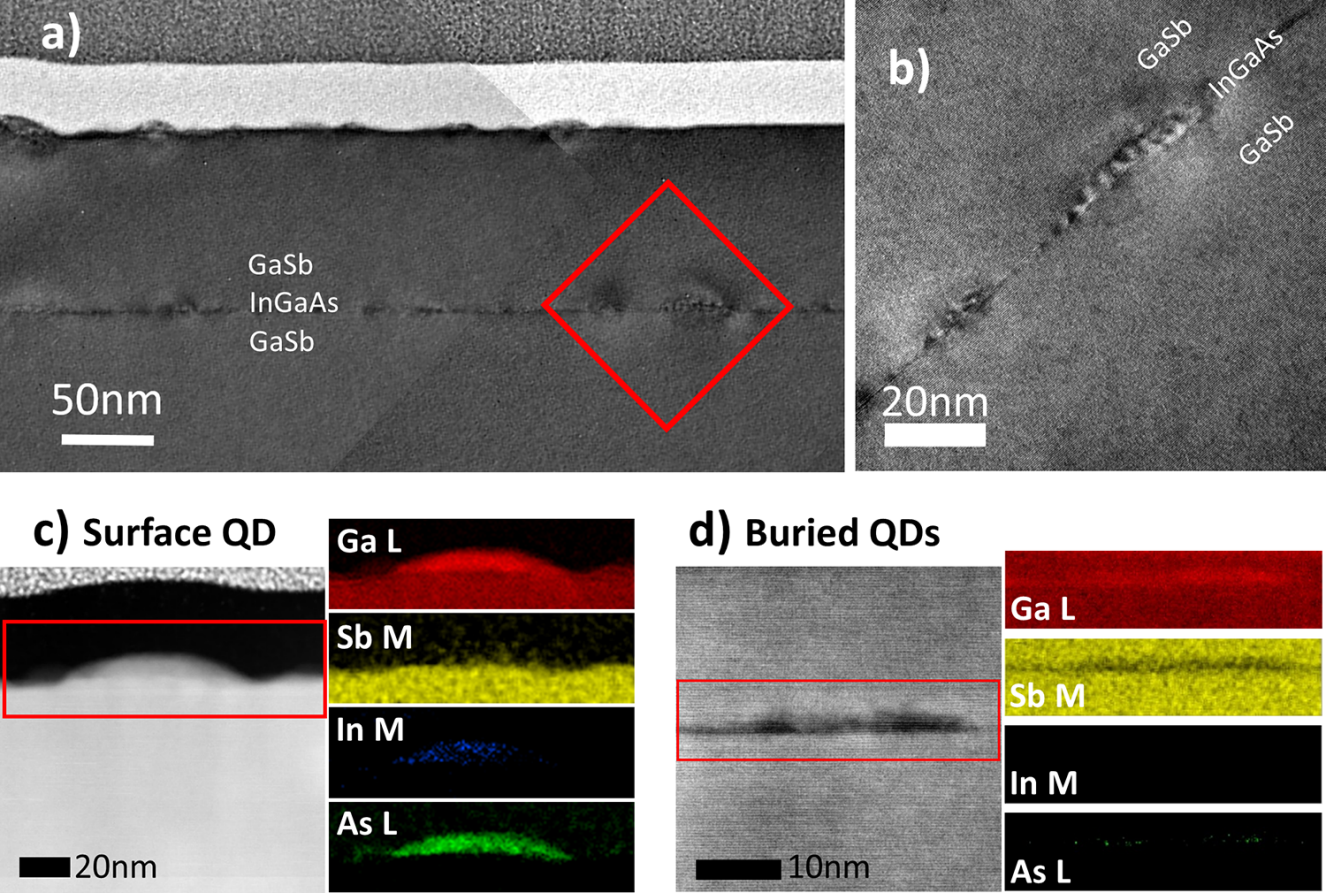
(a) BF TEM image montage of a 2 ML InGaAs QD layer surrounded by GaSb(111)A barriers, with a second layer of identical InGaAs QDs deposited at the surface. (b) BF TEM image of several buried 2 ML InGaAs QDs, corresponding to the red square in (a). ADF STEM image and corresponding EELS mapping signals of (c) a surface 2 ML InGaAs QD and (d) a buried 2 ML InGaAs QD.

### InGaAs(111)A Tensile-Strained QDs

#### QD Self-Assembly, Size, and Density

Figures 2(a)–(c) show how the reflection high-energy electron diffraction (RHEED) pattern evolves during growth of the sample structure in Figure 2(e). Prior to QD deposition, a streaky pattern (Figure 2(c)) indicates that homoepitaxial GaSb(111)A grown under the optimized growth conditions discussed above exhibits a smooth, 2D surface, consistent with Figure 1(d). From RHEED we determined a transition in the GaSb(111)A surface reconstruction at 425 °C from (2 × 4) to (12 × 1), in accordance with the findings of Proessdorf et al.^22^

Upon completion of the GaSb buffer, we insert a 10 s pause with no As_4_ or Sb_2_ fluxes (Figure 2(d)), to try and limit intermixing between the two group V species at the InGaAs/GaSb interface. As we initiate growth of the InGaAs, we see an abrupt change in the RHEED pattern from streaky to spotty, indicating the appearance of 3D QD nanostructures (Figure 2(b)). Then, once we switch the group V overpressure from As to Sb and grow several monolayers of GaSb, the streaky pattern returns, indicating a return to a 2D surface as the InGaAs QDs are buried (Figure 2(a)).

We found that three factors impacted the RHEED pattern evolution from Figure 2(b) to Figure 2(a), as well as the resulting sample morphology. First, it was helpful to maximize the amount of excess Sb available after InGaAs QD growth. We achieved this by halving the GaSb growth rate to 0.225 ML/s (compared to the 0.45 ML/s suggested in ref^23^) while keeping the Sb_2_ BEP constant at 3.1 × 10^–6^ Torr. Second, by increasing the InGaAs growth rate to 0.4 ML/s we could reduce the time for which the As was open, which also helped achieve a smooth GaSb top barrier more quickly. Third, we optimized the process for switching from III-As to III-Sb growth after the QDs. The 10 second pause step prior to starting growth of the GaSb top barrier in Figure 2(d) denotes a time delay that we introduced between closing the As shutter and cracker valve and then opening the Sb shutter (note that for flux stability the Sb cracker valve remains open throughout since the beam is efficiently blocked by the shutter alone).

For these specific growth conditions, we measured how long it took the RHEED to transition from the spotty pattern of the InGaAs QDs (Figure 2(b)) to the streaky pattern of the smooth GaSb(111(A) top barrier (Figure 2(a)). With a 0 s pause between closing the As and opening the Sb, we see a linear increase in the transition time with InGaAs coverage. This could be a function of the larger QDs that form, or simply how long the As is open. However, a 10 s pause between the As and Sb fluxes allowed us to reduce the RHEED transition time while optimizing surface morphology. If the critical pause is shorter, residual surface arsenic is still present when we initiate growth of the top GaSb top barrier. Longer critical pauses without an As flux could eventually destabilize the InGaAs episurface. That being said, previous atomic force microscopy (AFM) studies of InAlAs(111)A samples held at higher temperatures (≥530 °C) for 1 min showed no loss of surface quality due to re-evaporation of the group V element.^24,25^ In light of the above, our optimal conditions for InGaAs QD growth are *T*_sub_ = 450 °C, a growth rate of 0.4 ML/s, a V/III BEP ratio of ∼350, and a 10 s pause between closing the As and opening the Sb after the InGaAs deposition (Figure 2(d)).

The AFM images in Figures 3(a),(b) correspond to the 2D-to-3D RHEED transition shown in Figures 2(a),(b). As soon as we start to deposit InGaAs onto the smooth GaSb(111)A surfaces, we see the self-assembly of 3D QD nanostructures that grow larger and more dense as we increase InGaAs coverage (Figures 2(b)–(d)). We also see evidence of small pits, ∼1 nm deep, on the InGaAs surface (Figure 3(b)). Similar features have been observed previously for other ternary alloys grown on (111)A surfaces, even those where As is used throughout, and hence no group V interruption was needed.^26^ As a result, we do not believe the pits are caused by excessive group V detachment during the 10 s growth pause. Instead, consistent with those earlier studies,^26^ we attribute their formation to nanoscale phase segregation into slightly InAs- and GaAs-rich clusters, possibly exacerbated in this case by the presence of the tensile strain.

We analyze multiple AFM images from across the surface of each sample to compile statistics of QD height, diameter, and areal density as a function of InGaAs coverage (Figure 4). InGaAs QDs on the sample with 0.75 ML coverage (Figure 3(b)) have a mean diameter of 31.3 nm, a mean height of 2.9 nm (Figures 4(a) and (d)), and an areal density of 7.8 × 10^8^ cm^–2^ (Table 1). The self-assembly of QDs for InGaAs coverage < 1 ML is consistent with the Volmer–Weber (VW) growth mode as opposed to the more usual Stranski–Krastanov (SK) growth mode for which QD growth is preceded by the formation of a continuous wetting layer ≥ 1 ML thick. This is in keeping with previous reports of VW self-assembly in certain other tensile-strained QD systems including GaP/GaAs^12^ and Ge/InAlAs.^27^

**Table 1:** Mean QD Size and Areal Density as a Function of InGaAs/GaSb(111)A Coverage

InGaAs coverage (ML)	QD diameter (nm)	QD height (nm)	Areal density (×10^8^ cm^–2^)
0.75	31.3 ± 5.8	2.9 ± 0.7	7.8
1.0	46.0 ± 12.2	4.0 ± 0.8	6.5
2.0 (large)	69.2 ± 3.7	7.1 ± 1.5	6.0
2.0 (small)	26.2 ± 10.4	3.7 ± 1.0	160

As we raise InGaAs coverage to 1 ML (Figure 3(c)), the average diameter and height of the QDs increase (Figures 4(b) and (e)), but their areal density remains essentially constant (Table 1). Doubling the InGaAs coverage to 2 ML causes another change in the surface morphology (Figure 3(d)). A population of even larger QDs with a similar areal density to Figures 3(b),(c) coexists with a second population of smaller QDs, whose areal density is ∼20× higher (Table 1). The histograms in Figures 4(c) and (f) confirm this bimodal distribution of QD diameters and heights for the 2 ML InGaAs sample.

From Figures 3 and 4, it seems that self-assembly begins with an initial formation of low-density QDs via the VW growth mode. These QDs grow larger as the InGaAs deposition amount increases but their density does not change. However, at some point between 1–2 ML InGaAs coverage, we see the nucleation of a second population of much higher density QDs.

#### InGaAs QD Structure, Strain, and Composition

We used transmission electron microscopy (TEM) to examine the internal structure and crystallinity of the InGaAs/GaSb(111)A samples, specifically looking for evidence of defects originating from the QD layer. Figure 5(a) is a montage of BF TEM images from a 1 ML InGaAs/GaSb(111)A sample. Strain contrast in the InGaAs means that the QDs show up as dark spots across the width of the image. We see no evidence of threading dislocations or other crystallographic defects originating at the tensile-strained InGaAs layer in this montage. Figure 5(b) shows a higher magnification image of three InGaAs QDs from the red square in Figure 5(a), while Figure 5(c) shows a high-resolution high-angle annular dark field (HAADF) scanning TEM (STEM) image of an individual InGaAs QD from the left side of Figure 5(b). The absence of dislocations or other defects in these images suggests that these 1 ML InGaAs QDs are fully strained to the surrounding GaSb(111)A matrix.

Figure 6(a) is a montage of bright-field (BF) TEM images from a 2 ML InGaAs/GaSb(111)A QD sample. As for the 1 ML QD sample, the tensile strain appears to be closely confined to the darker 2 ML InGaAs layer. In contrast with the 1 ML QDs, here we see a low density of threading dislocations originating at the 2 ML InGaAs QD layer.

We see evidence of both large and small InGaAs QD populations in Figure 6(a), consistent with AFM from this sample (Figure 3(d)). Strain contrast around the smaller 2 ML InGaAs QDs in Figure 6(a) is very similar to that for the 1 ML QDs in Figure 5, suggesting that they are fully strained to the GaSb matrix. However, this does not appear to be the case for the population of larger 2 ML QDs. A higher magnification BF TEM image of two of these larger InGaAs QDs reveals a distinctive striped contrast pattern (Figure 6(b)).

One explanation for this striped pattern could be a series of periodic defects through the QD, similar to the interfacial misfit arrays known to form in certain strained heteroepitaxial systems, such as GaSb grown on GaAs(001).^28^ Alternatively, the striped pattern could be Moiré fringes due to interference between the different lattice constants of the InGaAs and GaSb. Moiré fringes would therefore suggest that these QDs are strain relaxed. When we perform fast Fourier transform filtering on higher magnification images, we do see evidence of a second, smaller lattice constant matched to the QD location. Regardless of whether it is due to contrast from periodic defects or Moiré fringing, the presence of this striped pattern suggests at least partial relaxation of the tensile strain in the larger 2 ML InGaAs QD population.

Figure 6(c) shows an ADF STEM image and corresponding electron energy loss spectroscopy (EELS) maps of a surface QD. As expected, the In, Ga, and As EELS signals confirm that the QD is composed of InGaAs. The In and As signals are well confined to the QD, suggesting that our strategy to limit arsenic attacking the GaSb(111)A surface prior to InGaAs growth (Figure 2(d)) has been successful. Interestingly, however, we can also observe the presence of Sb inside the quantum dots. Due to its surfactant nature, Sb is well-known to surface segregate during III-Sb growth. This effect, combined with the large Sb/Ga BEP ratio we used for growth of the GaSb(111)A barriers (see above), means that an excess of Sb was likely available for unintentional incorporation into the growing InGaAs QDs. A previous study of InAs/GaAs(001) QDs grown in the presence of an Sb flux shows that ∼25% of the As atoms were replaced with Sb.^29^ Interestingly, the authors noted this effect only in the wetting layer where the strain was lower than the ∼7.2% in the QDs. Hence, given that the InGaAs/GaSb system in this work has a strain of ∼4%, it is perhaps unsurprising that we similarly observe Sb incorporation.

Figure 6(d) shows an equivalent ADF STEM image and EELS maps of a buried InGaAs QD. Comparison with Figure 6(c) reveals a clear flattening of the buried QDs as a result of capping with GaSb. This change of shape during the capping process is well-known in other QD systems and takes place due to atomic diffusion.^30^ The EELS maps show that although Sb content in the buried InGaAs QDs is lower than in the surrounding GaSb matrix, it is nevertheless present. We note that although the As EELS signal is barely visible in the buried InGaAs QD, it must be there. This apparent absence is likely due to the overlap of As with the Ga edge (observe the increased Ga content of the InGaAs QD with respect to the GaSb matrix). This issue demonstrates that using EELS for quantitative compositional analysis of these QDs would be challenging. Even so, it seems reasonable to claim a qualitative reduction in As content in the buried QD layer compared to the surface QDs. From this we can infer that the buried QDs have higher Sb content than those on the surface; to maintain stoichiometry, As leaving a QD must be replaced by Sb diffusing in.

It is important to note that previous studies of In_0.5_Ga_0.5_As deposited on GaSb(001) reported the appearance of a spotty RHEED pattern during growth (as per Figure 2(b)), suggesting 3D QD formation.^17^ However, *ex situ* TEM analysis showed that the InGaAs/GaSb(001) QDs had subsequently undergone a 3D-to-2D transformation into smooth QWs, which the authors attributed to the surfactant effects of excess Sb at the surface. That postgrowth AFM (Figure 3) and TEM (Figures 5 and 6) show clear evidence of InGaAs/GaSb(111)A QDs in our samples suggests that the group III termination of the (111)A surfaces limits the Sb surfactant effects that are so pronounced on (001).

#### Interband QD Light Emission

**Figure 7:**
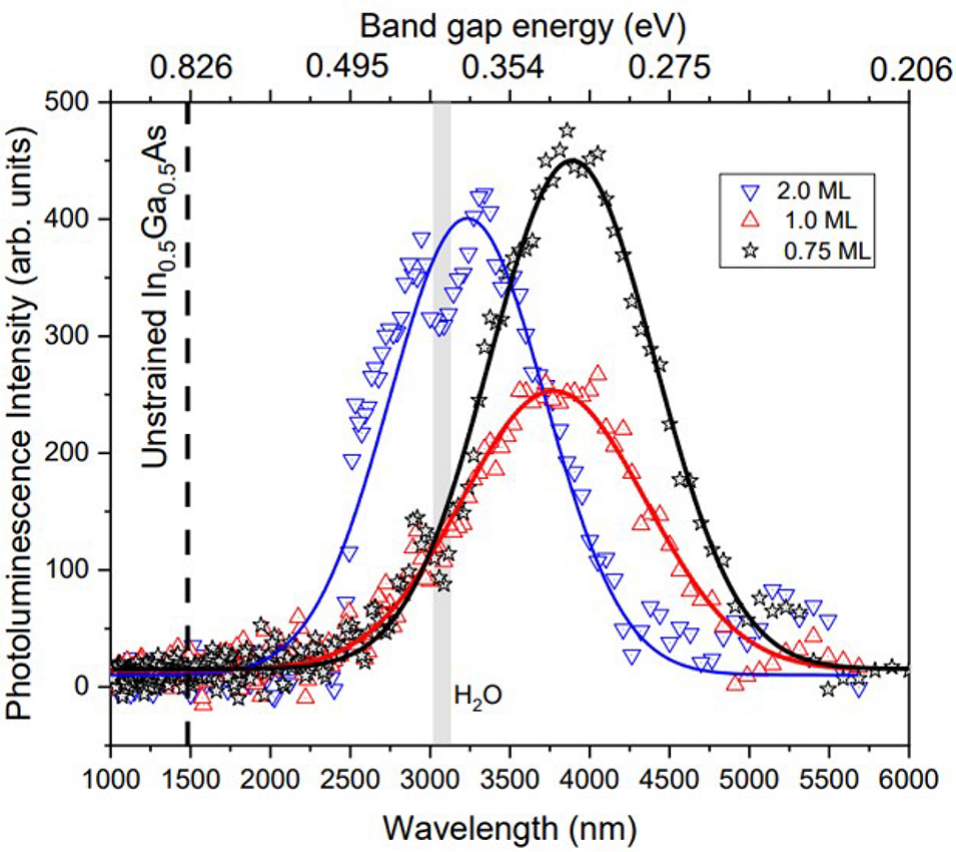
PL spectra at 77 K from InGaAs/GaSb(111)A samples containing a single layer of 0.75, 1.0, and 2.0 ML QDs. The continuous lines are Gaussian fits to each spectrum. The gray line highlights the position of a water absorption feature present in all three spectra at ∼3000 nm.^31^

Tensile-strained InGaAs/GaSb(111)A QDs are optically active, in contrast with the InGaAs/InAs(111)A QDs we explored in an earlier study.^32^ PL measurements reveal InGaAs QD light emission at wavelengths of 3889, 3773, and 3233 nm for the 0.75, 1.0, and 2.0 ML samples, respectively (Figure 7). In each case, we verified that this emission was from the InGaAs QDs by comparing the QD samples to PL from bulk GaSb(111)A control samples (Figure 1(f)). We also used 2200 nm long-pass filters to rule out harmonic emission from the GaSb barriers as the origin. PL intensity from these single layers of InGaAs QDs is somewhat weak, which is likely a function of their type-II and type-III band alignments. To enhance light emission intensity for future device applications, we will explore stacking multiple InGaAs QD layers, placing the QDs closer to the surface and incorporating Al(Ga)Sb barriers to improve carrier confinement in the active region.

We note some interesting features in these PL spectra (Figure 7). First, and perhaps most striking, is the large red-shift in the emission wavelength for the tensile-strained InGaAs QDs (3233–3889 nm) compared to 1480 nm for bulk In_0.5_Ga_0.5_As at 77 K (dashed line in Figure 7).^33^ This wavelength increase of ≥1750 nm indicates that the band gap reduction induced by the ∼4% tensile strain has a greater impact on QD electronic structure than quantum confinement effects that lift the electron ground state above the conduction band minimum (CBM).

Table 2 compares PL emission from our In_0.5_Ga_0.5_As(111)A QDs to the few existing reports of light emission from tensile-strained (In)GaAs(001) QDs and QWs. Those reports all described PL emission at wavelengths from 1.7 to 3.0 μm. By creating defect-free, tensile-strained In_0.5_Ga_0.5_As QDs we can achieve light emission deeper into the IR than anything previously reported.

**Table 2:** Light Emission from In(Ga)As Nanostructures on GaSb

Nanostructure	Substrate	Wavelength (μm)	Ref
In_0.5_Ga_0.5_As QW	GaSb(001)	1.77–3.00	(17)
In_0.5_Ga_0.5_As QW	GaSb(001)	2.09–2.25	(18)
GaAs QDs on GaAsSb	GaSb(001)	1.91–2.25	(19)
GaAs QDs	GaSb(001)	∼1.91	(20)
In_0.5_Ga_0.5_As QDs	GaSb(111)A	3.23–3.89	present work

The second feature of note in Figure 7 is that as we increase InGaAs deposition from 0.75 to 2.0 ML, we see an unusual response in the QD emission wavelength. Quantum size effects dictate that the confined electron ground state of a QD will decrease in energy as the QD gets larger, red-shifting the light emission. However, as we increase the InGaAs coverage and the QDs get larger (Table 1), we see that the PL *blue-shifts* to shorter wavelengths.

**Figure 8:**
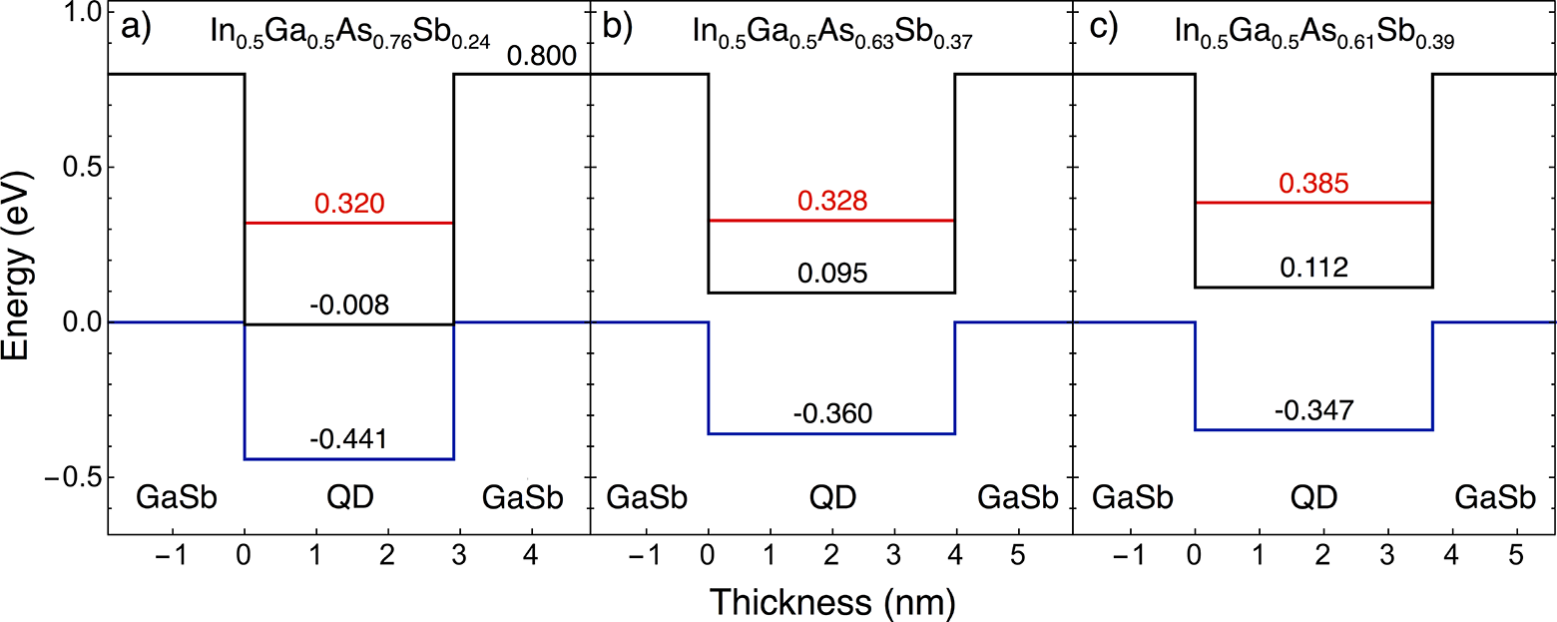
Calculated band alignments at 77 K for (a) 0.75 ML, (b) 1.0 ML, and (c) 2.0 ML InGaAs(Sb)/GaSb(111)A QDs. A type-III band alignment exists for the 0.75 ML QDs, with a type-II alignment for the 1.0 and 2.0 ML QDs. The CBM is shown in black, and the VBM in blue. Red lines indicate the electron ground state energies.

To understand this unexpected result, we used a computational band structure model that accounts for bulk band gap, tensile strain, and quantum confinement. These parameters vary interdependently as we adjust QD composition and size. We have described this model previously.^16,32^ To obtain agreement between the calculated electron–hole radiative recombination energies and the experimental PL data, we found that we needed to include a certain amount of Sb in the nominally In_0.5_Ga_0.5_As QDs (Figure 8). This change to the QD composition is justifiable based on the EELS results in Figures 6(c),(d). We express the corresponding band parameters of the resulting quaternary InGaAsSb alloy as a weighted sum of the related ternary values.^33^ According to the model, unintentional Sb incorporation results in In_0.5_Ga_0.5_As_1–*y*_Sb_*y*_ QDs with *y* = 0.24, 0.37, and 0.39 for the 0.75, 1.0, and 2.0 ML samples, respectively.

Our calculations show that 0.75 ML QDs have a type-III band alignment: the CBM of the InGaAs(Sb) QDs and valence band maximum (VBM) of the GaSb(111)A barriers overlap (Figure 8(a)). If the QDs are too large, the electron ground state will lie close to the InGaAs CBM, hence allowing the quasi-bound electron to escape to the GaSb VBM through an interband tunneling process.^34,35^ However, if the QDs are small enough, quantum confinement lifts the electron ground state above the InGaAs(Sb) CBM, to open up an effective band gap. For this type-III band alignment, there is therefore a critical QD size for bound or quasi-bound states to appear in the InGaAs(Sb). Using experimentally derived values for QD diameter and height (Table 1), we calculate that the average 0.75 ML In_0.5_Ga_0.5_As_0.76_Sb_0.24_/GaSb QD is below this critical size, and hence hosts a bound electron state (Figure 8(a)). Our model predicts that electron–hole recombination between the QD ground state and the GaSb VBM will produce photons with λ = 3875 nm (0.320 eV). This result agrees with the PL emission from the 0.75 ML sample at λ = 3889 nm (0.319 eV) in Figure 7.

Our model shows that a 1.0 ML QD of average size (Table 1) has a type-II band alignment, where a gap exists between the CBM of the In_0.5_Ga_0.5_As_0.63_Sb_0.37_ QD and the VBM of the GaSb(111)A (Figure 8(b)). Our model predicts photon emission at λ = 3780 nm (0.328 eV), in good agreement with the experimental value of λ = 3773 nm (0.329 eV) in Figure 7.

It is interesting that despite the bimodal size distribution of the 2.0 ML QDs, PL from this sample shows only a single peak, suggesting emission from only one of the two QD populations. (Note that the apparent dip at ∼3000 nm in all three spectra in Figure 7 is due to absorption by water in the beam path.^31^)

We therefore used our model to perform a band alignment calculation for both the large and small QD populations we observed in AFM (Figures 3 and 4). Using the average QD size for the population of larger QDs (Table 1), we were unable to find a reasonable solution that agreed with the PL emission measured for the 2.0 ML sample. However, we have seen that Figure 6 shows evidence of strain relaxation in larger QDs in the 2.0 ML sample. Dislocations and other strain-relieving defects typically act as centers for nonradiative recombination, which is consistent with the lack of light emission from this population of larger QDs.

If we instead base our calculation on the average size of the smaller 2.0 ML QD population (Table 1), we obtain the band alignment shown in Figure 8(c). We again see a type-II band alignment with a confined electron state in the In_0.5_Ga_0.5_As_0.61_Sb_0.39_ QD. The model predicts photon emission at λ = 3221 nm (0.385 eV), in good agreement with the experimental value of λ = 3233 nm (0.384 eV) in Figure 7.

It is interesting that our model suggests lower Sb incorporation for the 0.75 ML QDs compared with the 1.0 and 2.0 ML QDs, although this could simply be a function of the additional time it takes to grow the QDs with higher InGaAs coverage. In the future it might be interesting to explore whether we can control the incorporation of Sb into the InGaAs QDs. We know from the work of Taliercio et al. that surface Sb can have a profound effect on InGaAs/GaSb(001) nanostructure formation.^17^ Limiting the amount of excess Sb available at the underlying GaSb(111)A surface might therefore be beneficial. Techniques that could help with this include a higher growth rate for the GaSb(111)A bottom barrier to lower the V/III flux ratio, a longer pause without Sb flux, or even a flash exposure of the GaSb(111)A surface to Ga to react away any surface Sb before starting InGaAs growth.

## Conclusions

This paper describes a reproducible set of conditions for the MBE growth of high-quality homoepitaxial GaSb(111)A with extremely smooth surface morphology. The deposition of In_0.5_Ga_0.5_As onto these GaSb(111)A surfaces results in the self-assembly of QDs under ∼4% tensile strain. One can tailor QD diameter, height, and areal density via the InGaAs coverage. Below some threshold size, the InGaAs(111)A QDs are fully strained and free from defects. Band structure modeling shows that the QDs exhibit a type-II or type-III band alignment depending on their size. Along with EELS analysis, these models also suggest unintentional incorporation of Sb into the InGaAs QDs. Due to the residual tensile strain, the InGaAs(Sb)/GaSb(111)A QDs exhibit band-to-band PL emission that extends into the mid-IR at λ = 3.2–3.9 μm. The ease of synthesis and wavelength tunability afforded by tensile-strained QDs are attractive attributes that will benefit efforts to create next-generation mid-IR optoelectronic devices.

## Methods/Experiments

All samples are grown using solid-source MBE on unintentionally doped (i.e., p-type), nominally on-axis , 500 μm thick GaSb(111)A substrates. We use indium to mount the GaSb(111)A substrates onto molybdenum blocks to ensure temperature uniformity. We monitor substrate temperature (*T*_sub_) using a BandiT temperature monitoring tool and a thermocouple located behind the substrate, calibrated by RHEED against known reconstructions of the GaSb(001) surface. We select InGaAs composition via the InAs and GaAs growth rates in monolayers per second (ML/s), calculated from RHEED intensity oscillations and adjusted for the atomic density of the GaSb(111)A surface. We used valved cracker cells for the group V species, configured to provide As_4_ and Sb_2_.

The general sample structure consists of a 250 nm GaSb(111)A buffer that serves as a bottom barrier for the InGaAs QDs. We then deposit 0.75 to 2.0 ML of InGaAs to form the tensile-strained QDs. For PL measurements we cover the QDs with a 75 nm GaSb top barrier and finally deposit an identical layer of InGaAs QDs on the surface for AFM studies (Figure 2(e)).

Our MBE growth conditions are based on the few existing studies of GaSb(111)A homoepitaxy and InGaAs/InAs(111)A QDs.^23,36^ We use a constant substrate temperature of *T*_sub_ = 450 °C for growth of both the GaSb barriers and InGaAs QDs. Sample morphology is highly sensitive to the Sb_2_ flux. The sticking coefficient of group V atoms is known to be lower on (111)A surfaces than (001) surfaces.^37^ In addition, when growing heteroepitaxial III-As and III-Sb materials, it is important to take into account the strong propensity for anion exchange. Since Ga–As bonds are stronger than Ga–Sb bonds, there is a thermodynamic driving force for As atoms to replace Sb atoms in the episurface.^38^ We therefore explored Sb_2_ BEPs of 1.5–3.1 × 10^–6^ Torr and GaSb growth rates of 0.225–0.45 ML/s to optimize the V/III flux ratio. We also investigated how best to switch back and forth between Sb and As by introducing a pause between opening and closing the shutters of the two group V sources.

We studied the morphology of sample surfaces with AFM. A combination of cross-sectional BF TEM, BF and HAADF STEM, EELS, and energy dispersive X-ray spectroscopy (EDS) mapping by STEM allowed us to look at the internal structure, crystalline quality, and composition of the QDs. To analyze the optical quality of the samples we performed Fourier-transform infrared (FTIR) PL spectroscopy at 77 K using a 300 mW laser with an InSb detector and a 2200 nm long-pass filter to block any harmonic signals from the laser or GaSb barriers. We calculated tensile strain between the In_1–*x*_Ga_*x*_As alloy and GaSb as a function of the Ga composition to find the critical thicknesses for strain relaxation and for use in our band structure calculations. To calculate the confined states in the InGaAs QDs, we used a computational model based on that described in ref (16).
